# Socioeconomic disparities in Papanicolaou test utilization in Western Iran

**DOI:** 10.1186/s12889-024-17927-x

**Published:** 2024-02-14

**Authors:** Bahare Safari-Faramani, Roya Safari-Faramani, Farid Najafi, Davoud Khorasani Zavareh, Ali Kazemi Karyani, Mitra Darbandi

**Affiliations:** 1 Students Research Committee, Kermanshah University of Medical Sciences, Kermanshah, Iran; 2 Social Development and Health Promotion Research Center, Research Institute for Health, Kermanshah University of Medical Sciences, Kermanshah, Iran; 3 Department of Health in Emergencies and Disasters, Workplace Health Promotion Research Center (WHPRC), School of Public Health and Safety, Shahid Beheshti University of Medical Sciences, Tehran, Iran; 4 Department of Neurobiology, Care Sciences and Society (NVS), H1, Division of Family Medicine and Primary Care, Huddinge, Sweden; 5 Research Center for Environmental Determinants of Health (RCEDH), Health Institute, Kermanshah University of Medical Sciences, Kermanshah, Iran

**Keywords:** Cervical cancer, Inequality, Iran, Papanicolaou test, Socioeconomic status

## Abstract

**Background:**

Cervical cancer remains the fourth most frequently diagnosed cancer among women, and its impact is particularly significant in women residing in less developed countries. This study aims to assess socioeconomic inequality in using Papanicolaou tests, commonly known as Pap tests, which are crucial for detecting cervical cancer. The research also seeks to decompose this inequality, identifying its contributing factors. This investigation is conducted within a sizable population-based study focused on the Kurdish population, with an additional examination of potential variations between urban and rural areas.

**Method:**

The study utilized baseline data from the Ravansar Non-Communicable Disease Cohort Study (RaNCD), involving 3,074 ever-married women aged 35–65. Asset data was employed to determine socioeconomic status (SES), and Principal Component Analysis was applied. The uptake of Papanicolaou tests was assessed for inequality using the Concentration Index (Cn). Additionally, decomposition analysis was conducted to identify and understand the factors contributing to socioeconomic inequality.

**Results:**

The study found that overall, 86% of women reported having undergone cervical cancer screening at least once in their lifetime. The Concentration Index (Cn) for the total population was 0.21 (*p* < 0.0001), indicating a higher concentration of Papanicolaou test uptake among wealthier groups. In urban areas, the Cn was 0.34 (*p* < 0.0001), reflecting a significant concentration among the rich. However, in rural areas, the Cn was -0.10 (*p* = 0.3006), suggesting no significant socioeconomic inequality. Factors such as socioeconomic status (SES), education, and age contributed to reducing inequality, explaining 62.7%, 36.0%, and 1.7% of the observed inequality, respectively. Interestingly, place of residence had a negative influence on inequality.

**Conclusion:**

The uptake of Papanicolaou tests varies across different socioeconomic status levels, with a higher concentration among wealthier groups. The results enable health policymakers and researchers to tailor health intervention toward increasing public awareness, especially among women with lower levels of education women in economically deprived groups.

**Supplementary Information:**

The online version contains supplementary material available at 10.1186/s12889-024-17927-x.

## Background/introduction

Despite the availability of integrated human papillomavirus (HPV)-based screening and vaccination, cervical cancer continues to claim the lives of approximately 300,000 women and affects nearly 600,000 annually, with a higher impact on middle-aged women and those residing in lower-resource settings [1]. Globally, cervical cancer ranks as the fourth most commonly diagnosed cancer among women and stands among the top three cancers affecting women under the age of 45 in most regions [1, 2]. Unfortunately, women in less developed countries bear a disproportionately greater burden, highlighting cervical cancer as a disease that particularly afflicts the economically disadvantaged [1, 3].

The primary causative factor for cervical cancer is the sexually transmitted human papillomavirus (HPV), which not only poses a health risk but also subjects affected women to potential stigmatization. This dual impact further impedes access to health services, exacerbating the toll of cervical cancer on affected populations [3].

The Papanicolaou cervical screening method has been instrumental in preventing a substantial proportion of cervical cancers since its introduction [4]. While widely recommended for detecting precancerous lesions [5], its effectiveness has been less pronounced in lower-income countries [6]. Reports from various regions illustrate disparate uptake rates, such as less than 5 percent in Uganda [7], around 31 percent in Jordan [8], approximately 27 percent in Malaysia [9], and 35 percent among Lebanese women [10]. Notably, rates range from less than 30 percent in Georgia, Azerbaijan, Tajikistan, and Uzbekistan to approximately 100 percent in Finland [6].

In Iran, the scenario is challenging. A national survey disclosed that only half of women aged 30–59 years have ever undergone the Papanicolaou test for cervical cancer screening [11]. Regionally, the uptake of cervical screening is reported as 32 percent in Kurdistan (western part of Iran) [12] and around 28 percent in Ardebil [13]. Despite the widespread availability of cervical cancer screening services through Primary Health Care in Iran, limited evidence suggests lower uptake in disadvantaged subgroups [14]. Post-menopausal and women with lower levels of education reportedly derive fewer benefits [12, 14–16], and residence in rural areas is associated with lower uptake [11, 15, 16].

In Iran, the cervical cancer screening program was initiated in 1989. Initially, it targeted women aged 20 to 65, and as of 2017, the focus has shifted to high-risk women aged 35 to 59. The latest guidelines from the Ministry of Health and Medical Education recommend the Papanicolaou test for married women, particularly those who have been sexually active after the age of 30 or three years after initiating sexual activity. Following normal results, the screening continues at 5-year intervals. This screening service is integral to primary public health care (PHC) and is administered by midwives. Additionally, in private clinics, gynecologists and midwives offer these services upon request [11, 17, 18].

The study underscores the growing importance of examining variations in cervical cancer screening uptake across different socioeconomic strata. Despite the provision of free cervical cancer screening services in Iran, understanding the disparities in uptake among diverse social groups is crucial for effective planning and intervention strategies to address low participation rates. This research focuses on the impact of socioeconomic status on the utilization of Papanicolaou tests, aiming to quantify and dissect the socioeconomic inequality within the Kurdish population. The investigation is part of the Ravansar Non-Communicable Disease Cohort Study (RaNCD), a large-scale population-based initiative.

Up to our knowledge, evidence on Pro-rich inequality in the uptake of the Papanicolaou test is limited in Iran. The study's primary objectives involve measuring socioeconomic inequality in Papanicolaou test uptake and performing a decomposition analysis to identify factors contributing to this inequality within the Kurdish population. Additionally, the research aims to explore potential differences in screening uptake between urban and rural areas.

## Method

### Study population

Baseline data from the RaNCD (Ravansar Non-Communicable Disease) cohort, one of the 19 centers of the Prospective Epidemiological Research Studies of IrAN (PERSIAN) cohorts, were utilized. PERSIAN is primarily a population-based cohort, and the study's methodology has been described in detail elsewhere [19–22]. The main study is a cohort, and the present analysis involves a cross-sectional examination of baseline data from the participants, which were collected in 2017.

Ravansar is one of the districts of Kermanshah Province. Its population is around 50,000, and the majority of its population is Kurdish. In this study, ever-married women aged 35–65 years residing in Ravansar for at least nine months of the year were invited to participate in the study.

The RaNCD study commenced in 2014, and the recruitment phase continued until 2017. The baseline data collected from 2014 to 2017 is utilized in this study. The duration of the follow-up for this cohort is 15 years.

In the baseline phase, 10,065 participants were recruited. Among them, 5,249 were women, and 326 of the women were single, so they were excluded from the present analysis. Of the 4,964 ever-married women, 3,074 answered the questions about the lifetime history of Papanicolaou tests, which is the outcome variable. For around 1,800 participants, the response to the question "Having ever had a pap smear" was missing. Therefore, the study sample for the analyses was restricted to those who responded to the related question. The final sample size, after removing the missing cases, was 3,074.

The authors inquired about the lifetime history of Papanicolaou tests, measuring it as yes or no. The exact question was: "Have you ever had a pap smear?" the response was dichotomous, categorized as either yes or no. Therefore, the outcome assessed was ever-screening in ever-married women aged 35–65 who participated in the RaNCD study in 2017 [19].

The age of the participants was measured based on their age at the time of the interview and then categorized into three groups: 35–44, 45–54, and 55–65 years old. Based on self-report, marital status included categories for married, widowed, or divorced individuals. The education level of participants was categorized as no formal schooling, elementary, middle, and high school diploma, and higher education, including university degrees. The residence was categorized as living in urban or rural areas of Ravansar City.

To assess inequality, socioeconomic status (SES) was defined based on household asset data using Principal Component Analysis (PCA). Items included house area per capita, room per capita, access to a freezer, washing machine, internet, motorcycle, car, vacuum cleaner, ownership of mobile, ownership of the internet, number of read books, number of foreign travels, number of non-pilgrimage foreign travels, and number of nation travels. PCA with Varimax rotation resulted in four components, and the first component was used to define the socioeconomic status rank [23] (Supplementary 1). The SES index was grouped into five quintiles: the 1st quintile represents the poorest group, and the 5th quintile represents the richest one. Dummy variables for age group, marital status, residency, educational level, and socioeconomic groups were defined.

### Statistical analysis

The Concentration index (Cn) is employed to evaluate inequality in utilizing the Papanicolaou test across the socioeconomic status (SES) spectrum. This index relies on the construction of a concentration curve [24]. The concentration curve depicts the cumulative percentage of a population based on their SES along the horizontal axis, juxtaposed with the cumulative percentage of the health outcome, specifically the uptake of the Papanicolaou test.

The Concentration index (Cn) is calculated as twice the area enclosed by the concentration curve and the line of equality, represented by the 45-degree diagonal line. The Cn value varies between -1 and + 1, signifying distinct patterns of socioeconomic-related inequality in the health variable under consideration. A Cn value of -1 indicates an unequal concentration of the health variable among the economically disadvantaged, while a + 1 signifies a concentration of the health outcome among the affluent. A Cn value 0 denotes the absence of socioeconomic-related inequality [24].

The authors used the following formula to measure C_n_:

$$\mathrm{Cn }=\frac{2 cov(yi ri)}{\pi }$$

The health outcome variable (yi), representing the uptake of the Papanicolaou test for participant i, and ri, the fractional rank of participant i in the distribution of the SES indicator, were used in the analysis. μ denotes the mean of the health outcome variable. As the outcome variable is binary, the authors normalized the Cn as:

$$\mathrm{Cn }=\frac{1}{1-\pi }$$

To identify the contributions of relevant factors to socioeconomic inequality, a decomposition of socioeconomic inequality in the uptake of the Papanicolaou test was performed (24).

As in a regression model where the outcome variable y is dependent on a set of k explanatory variables, x, the Concentration Index (Cn) for y can be decomposed as:

$$C={\sum }_{k}\left({\beta }_{k}{\overline{x}}_{k}/\mu \right){C}_{k}+{GC}_{\varepsilon }/ \mu$$

μ is the mean of the health outcome variable. In this study, the health outcome variable is the uptake of the Papanicolaou test, measured as 0 and 1. The mean of a dichotomous variable measured as 0 and 1 equals its prevalence.

Where k is the number of explanatory variables, ‾*xk*​​ is the mean of the explanatory variable, and Ck is the Concentration Index for each explanatory variable. The first component shows the contribution of the explanatory variable to the overall socioeconomic-related inequality. In the second component, *GCε*​ is the generalized Concentration Index for *ε* (25). This component indicates the proportion of socioeconomic inequality in the health outcome that the included explanatory variables do not explain. Data were analyzed using STATA version 14.2 (Stata Corp, Texas, USA) statistical software [25]. Finally to better subgroup analysis for place of residence (urban / rural) performed.

The Ethical Committee of the Research Deputy at Kermanshah University of Medical Sciences meticulously reviewed and approved the study under the assigned code KUMS.REC.1394.318. All participants provided informed consent before participating in the study, signifying their agreement.

## Results

Three thousand seventy four ever-married women aged 35–65 who expressed willingness to participate were enrolled in the study. Among these participants, 86% of ever-married women reported having undergone the Papanicolaou test at least once. Statistical analysis revealed a significantly elevated probability of Papanicolaou test uptake among various demographic subgroups, including younger age groups (*p* < 0.0001), married women (*p* < 0.0001), individuals residing in rural areas (*p* < 0.0001), those with higher educational attainment (*p* < 0.0001), and individuals belonging to higher socioeconomic strata (*p* < 0.0001) (Table 1).

**Table 1:** Characteristics of women 35–65 years old participated in the RaNCD study by Papanicolaou screening test behavior in 2017 (*n* = 3074)

Variable	Total (%) *N* = 3074	Pap Smear test		*p*-value*
		No (%)	Yes (%)	
Age groups (years)				
35–44	1548	138 (8.91)	1410 (91.09)	< 0.0001
45–54	1052	159 (15.11)	893 (84.89)	
55–65	474	125 (26.37)	349 (73.63)	
Marital status				
married	2865	366 (12.77)	2499 (87.23)	< 0.0001
Widow/divorced	209	56 (26.79)	153 (73.21)	
Residency				
Urban	2038	386 (18.94)	1652 (81.06)	< 0.0001
Rural	1036	36 (3.47)	1000 (96.53)	
Education level				
No formal schooling	1775	325 (18.31)	1450 (81.69)	< 0.0001
Elementary School	849	76 (8.95)	773 (91.05)	
Middle School	216	14 (6.48)	202 (93.52)	
High School diploma and higher	234	7 (7.35)	227 (92.65)	
Socioeconomic status				
1^st^ (the lowest)	615	123 (20.00)	492 (80.00)	< 0.0001
2^nd^	614	98 (15.96)	516 (84.04)	
3^th^	615	82 (13.33)	533 (86.67)	
4^th^	614	76 (12.38)	538 (87.62)	
5^th^ (the highest)	614	43 (7.00)	571 (93.00)	

^*^results of Chi Square test

The concentration index for the entire population was calculated to be 0.21 (*p* < 0.0001), signifying a higher concentration of Papanicolaou test uptake among affluent groups, as indicated in Table 2. Specifically, the concentration index for the urban population was 0.34 (*p* < 0.0001), highlighting a pronounced concentration among urban residents. Conversely, for the rural population, the concentration index was -0.10 (*p* = 0.3006), suggesting no significant concentration pattern in this subgroup.

**Table 2:** Normalized concentration indices for Papanicolaou screening test behavior among women 35–65 years old participated in RaNCD study by place of residence, 2017(*n* = 3074)

Region	Number of observation	Concentration Index (SE)	*p*-value
Total	3072	0.21 (0.03)	< 0.0001
Urban	2036	0.34 (0.03)	< 0.0001
Rural	1036	-0.10 (0.09)	0.3006

**Fig. 1:**
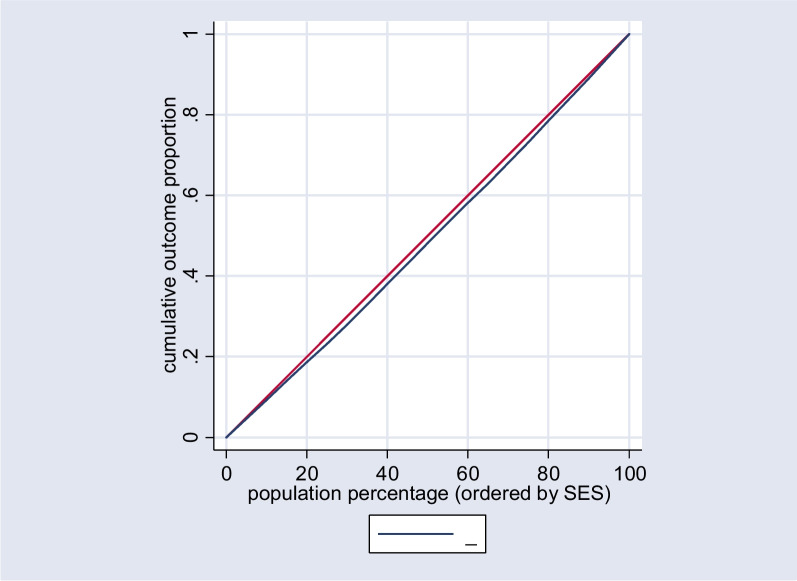
Concentration curve for the uptake of Papanicolaou tests in the total population. The red line is the equality line. The blue one shows the inequality in the total population

**Fig. 2:**
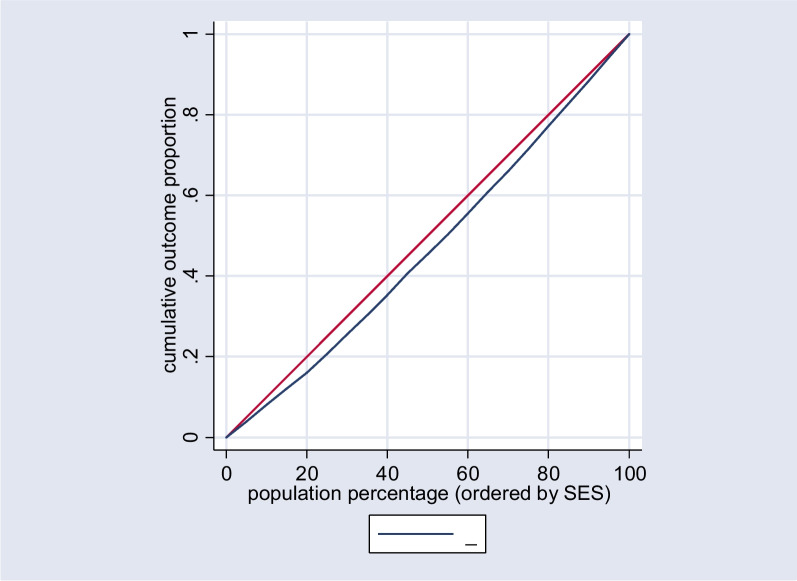
Concentration curve for the uptake of Papanicolaou tests in the urban population

**Fig. 3:**
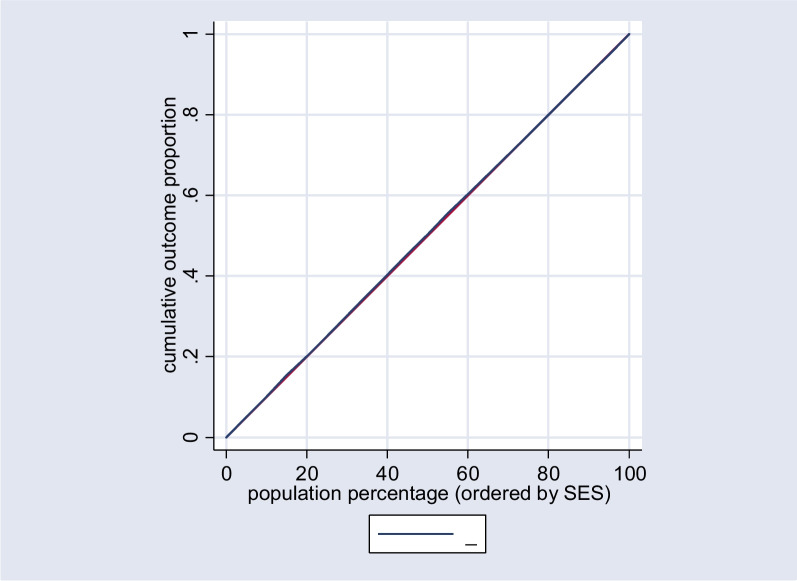
Concentration curve for the uptake of Papanicolaou tests in the rural population

A visual representation of these findings is provided in Fig. 1, which illustrates the concentration curves for the overall population. Additionally, Figs. 2 and 3 depict the concentration curves for the urban and rural populations, respectively.

Table 3 presents the decomposition of socioeconomic-related inequality in the uptake of the Papanicolaou test. The analysis reveals that SES emerged as the primary contributor to socioeconomic inequality, accounting for 62.7% of the observed inequality. Following SES, education, place of residence, and age contributed 36.0%, 2.2%, and 1.7%, respectively, to the observed inequality in Papanicolaou test uptake. Notably, place of residence exhibited a negative contribution, indicating a disproportionate concentration of rural individuals among the economically disadvantaged.

**Table 3:** Results of the decomposition analysis of socioeconomic inequality in Papanicolaou screening test behavior among women 35–65 years old participated in the RaNCD study, 2017 (*n* = 3074)

Variable	partial effect	proportion	Elasticity	ck	Absolute	percentage	%sum
Age groups (years)							
35–44	ref						
45–54	-0.0341542	0.34222511	-0.013654702	-0.01272537	0.001206675	0.70	1.700
55–65	-0.1146517	0.15419649	-0.020652909	-0.01222239	0.001752972	1.00	
Place of Residence							
Urban	ref						
Rural	0.0417949	0.33702017	0.016455285	-0.18406749	-0.02103391	-11.70	-2.2
Education level							
Illiterate	ref						
Elementary School	0.0666103	0.27618738	0.021491734	0.06660737	0.009941027	5.60	36.0
Middle School	0.0865794	0.07026675	0.007107071	0.24408194	0.012046582	6.70	
High School diploma and higher	0.1145206	0.07612232	0.010184081	0.60157506	0.042545066	23.70	
Socioeconomic status							
1^st^ (the lowest)	ref						
2^nd^	0.0599546	0.19986979	0.013998964	-0.39986975	-0.038873349	-21.60	62.7
3^th^	0.0820426	0.20019531	0.019187551	0.00032563	4.33892E-05	0.10	
4^th^	0.1508427	0.19986979	0.035220676	0.400521	0.097962642	54.50	
5^th^ (the highest)	0.0411641	0.19986979	0.009611519	0.80039075	0.053423407	29.70	
explained							98.200
residual							1.800
total							100.000

Interpreting the absolute contributions, a negative (positive) value implies that the socioeconomic inequality in Papanicolaou test uptake would increase (decrease) if that predictor were evenly distributed across the SES spectrum. Specifically, the negative contribution of place of residence suggests that socioeconomic inequality in Papanicolaou test uptake would intensify if the distribution of residence were uniform across SES categories.

The explained inequality in Papanicolaou test uptake was 0.2 (98.2% of Cn (0.21)), while the residual (unexplained) Concentration Index (Cn) was 0.003 (1.8% of Cn (0.21)), as detailed in Table 3.

## Discussion

This study aimed at assessing the inequalities in the uptake of the Papanicolaou test within a subset of the Kurdish population in the western region of Iran and investigate the factors contributing to this inequality. Among the 3,074 ever-married women surveyed, 86% had undergone the Papanicolaou test at least once, with a notably higher prevalence among individuals with higher socioeconomic status. The concentration index, calculated at 0.21, underscores a disproportionate Papanicolaou test uptake among affluent individuals.

Socioeconomic status emerged as the primary contributor, explaining approximately 63% of the observed inequality. Education followed as the second most influential factor. Notably, in the subgroup analysis, no significant inequality in the uptake of the Papanicolaou test was observed in rural areas.

Cervical cancer screening services are globally endorsed as an effective measure for preventing the second most common cancer in women. However, the utilization of these services exhibits significant disparities worldwide, with lower-income countries having a smaller share of coverage [6]. Screening coverage varies widely, with rates reported at approximately 78% in Spain and England, 98% in Finland, 65.6% in Korea, and 62% in Botswana [15, 16, 26–28]. In the current study, 86% of participants reported having undergone cervical cancer screening at least once in their lifetime, a notably higher prevalence compared to national reports, which indicate a screening rate of 52% for Iranian women and around 45% for women residing in Kermanshah Province [11].

It is crucial to acknowledge methodological differences between the present study and previous research, particularly the exclusion of single women in the present study. Given that the Papanicolaou test is integrated into Primary Health Care services (PHC), it predominantly caters to ever-married women. Consequently, the present study specifically directed questions about the history of Papanicolaou test participation to ever-married women.

Profound socioeconomic disparities in the uptake of the Papanicolaou test have been underscored across diverse global regions. Nunes MF et al. noted that, despite the widespread prevalence of cervical cancer screening, adherence to screening programs was notably lower among unemployed individuals, those with lower educational attainment, and women with lower incomes [29]. Also studies conducted across the European countries highlighted educational and income gradients in cervical cancer screening [30, 31]. Douglas et al. emphasized that cervical cancer screening test coverage was diminished among socioeconomically disadvantaged groups [27]. In Botswana, a concentration index as high as 0.2443 indicates that screening efforts were more concentrated among women with higher socioeconomic status (SES) than more disadvantaged [16]. Pro-rich inequality in Papanicolaou test uptake has also been documented in Korea and Peru [15, 26].

Addressing the complexities of inequality is undeniably intricate, and it is evident that a thorough understanding of the multiple underlying drivers is essential. Equipped with insights into these associated factors, more effective interventions can be strategically planned. There is a clear imperative for more comprehensive studies. The present study's findings align with those of Keetile M et al., whose research in Botswana demonstrated that wealth status was the primary contributor to observed inequality in Papanicolaou test uptake [16]. This consistency in results underscores the significance of socioeconomic status as a crucial factor in shaping inequalities in cervical cancer screening.

In Iran, the provision of the Papanicolaou test is facilitated through Primary Health Care (PHC) services and is offered free of charge. Notably, rural areas in Iran benefit from comprehensive coverage by the public sector, whereas the private sector is more active in urban areas. The current study reveals that inequality in Papanicolaou test uptake is evident in urban populations, while no such disparity exists in rural areas. This suggests that implementing population-based screening programs through the public health sector may effectively address this inequality [32].

Reports from studies conducted across Iran indicate that the knowledge, practices, and health literacy of women regarding cervical cancer are not satisfactory [33, 34]. Therefore, to enhance Papanicolaou test uptake in Iran, improvements in access need to be complemented by initiatives aimed at enhancing public awareness. It is crucial to underscore that achieving these objectives requires strong political commitment to ensure such interventions' successful implementation and sustainability.

This study is one of the most significant investigations conducted within a subset of Kurdish women, boasting a commendable sample size. A noteworthy strength lies in recruiting an equal sample size from rural and urban areas, enhancing the representativeness of the study population. Using trained interviewers following a standardized data collection protocol further ensures the gathered information's reliability and consistency. However, it is essential to acknowledge certain limitations inherent to the study.

One notable limitation involves the absence of information regarding the insurance status of the participants. The study's cross-sectional design also poses inherent limitations in establishing causal relationships. Given the nature of the study, it is imperative to exercise caution in drawing definitive conclusions about causality.

This study, like others published in Iran, focused exclusively on ever-married women, a demographic choice consistent with the characteristics of the national population, where approximately 90 percent of participants in a nationwide study fell into the ever-married category [11, 35–37]. However, it is crucial to acknowledge and critically assess the response rate in this study, which was reported at 70%. A comparison of demographic characteristics between participants and non-participants revealed significant differences, with participants generally being older, women with lower levels of education, predominantly residing in rural areas, and belonging to more disadvantaged groups. While the 70% response rate is commonly regarded as a critical threshold in research, the observed variations between participants and non-participants may introduce selection bias [38, 39], the direction of which remains unclear. Notably, non-response was primarily associated with a lack of public awareness about the Pap smear test due to the inclusion of predominantly rural and women with lower levels of education in the RaNCD study.

The reliance on self-reporting for collecting the history of Papanicolaou test uptake introduces a potential source of recall bias. However, efforts were made to minimize this bias by conducting face-to-face interviews in a secluded setting, allowing participants ample time for reflection. Moreover, it is noteworthy that ever-screening is considered less prone to recall bias compared to asking about a specific period, as highlighted by Jolidon V et al. [40]. Despite these considerations, the study underscores the importance of policies aimed at increasing public awareness and fostering a screening culture in society [41] to address barriers associated with knowledge gaps and promote regular screening practices [42, 43].

## Conclusion

Cervical cancer exerts a substantial impact on public health, standing as the second most common cancer among women. The Papanicolaou test, with its potential to prevent numerous cases of cervical cancer, serves as a critical tool in this context. This study sought to quantitatively assess the existing socioeconomic inequality in the uptake of the Papanicolaou test within a subset of the Kurdish population.

The study's findings indicate a discernible disparity in Papanicolaou test uptake across various levels of socioeconomic status, with a clear preference for higher socioeconomic groups. This discrepancy underscores the limited utilization of screening programs among economically disadvantaged individuals, where factors such as poverty and constrained access to healthcare play a significant role.

This evidence carries valuable implications for health policymakers and researchers. Tailoring health interventions innovatively, such as increasing public awareness, particularly among women with lower levels of education in economically deprived groups, is warranted. In light of the current evidence, further investigation is essential to identify the most impactful public health actions that have successfully mitigated health inequality in Papanicolaou test uptake.

### Supplementary Information

**Additional file 1:** **Supplement 1.** Results of Principal Component Analysis. **Table S1.** Communalities for asset variables collected in RaNCD study in 2017. **Table S2.** Total Variance Explained by the components based on Principal Component Analysis using Varimax rotation on asset data in RaNCD study 2017.

## Data Availability

The datasets generated during this study are available from the correspondence author on reasonable request via email.
