# Supplementary material for: Socioeconomic disparities in Papanicolaou test utilization in Western Iran
Source: BMC Public Health. 2024 Feb 14;24:471. doi: 10.1186/s12889-024-17927-x (PMC10868049; doi:10.1186/s12889-024-17927-x)
Supplement: Supplementary file 1 — Additional file 1: Supplement 1. Results of Principal Component Analysis. Table S1. Communalities for asset variables collected in RaNCD study in 2017. Table S2. Total Variance Explained by the components based on Principal Component Analysis using Varimax rotation on asset data in RaNCD study 2017. [file 12889_2024_17927_MOESM1_ESM.docx]

Supplement 1: Results of Principal Component Analysis

Table S1, Communalities for asset variables collected in RaNCD study in 2017

| Items | Initial | Extraction |
| --- | --- | --- |
| House area per capita | 1.000 | .837 |
| Room per capita | 1.000 | .829 |
| Have freezer | 1.000 | .398 |
| Have washing machine | 1.000 | .478 |
| Have internet | 1.000 | .417 |
| Have motorcycle | 1.000 | .143 |
| Have Car | 1.000 | .342 |
| Have vacuum cleaner | 1.000 | .364 |
| Ownership of mobile | 1.000 | .216 |
| Ownership of Internet | 1.000 | .524 |
| Number of read books | 1.000 | .403 |
| Number of foreign travels | 1.000 | .991 |
| Number of non-pilgrimage foreign travels | 1.000 | .990 |
| Number of nation travels | 1.000 | .271 |

Table S2, Total Variance Explained by the components based on Principal Component Analysis using Varimax rotation on asset data in RaNCD study 2017

| **Component** | **Initial Eigenvalues** | | | **Extraction Sums of Squared Loadings** | | | **Rotation Sums of Squared Loadings** | | |
| --- | --- | --- | --- | --- | --- | --- | --- | --- | --- |
|  | **Total** | **% of Variance** | **Cumulative %** | **Total** | **% of Variance** | **Cumulative %** | **Total** | **% of Variance** | **Cumulative %** |
| 1 | 2.443 | 17.451 | 17.451 | 2.443 | 17.451 | 17.451 | 1.984 | 14.175 | 14.175 |
| 2 | 1.912 | 13.658 | 31.109 | 1.912 | 13.658 | 31.109 | 1.771 | 12.648 | 26.823 |
| 3 | 1.592 | 11.370 | 42.479 | 1.592 | 11.370 | 42.479 | 1.741 | 12.432 | 39.255 |
| 4 | 1.257 | 8.978 | 51.457 | 1.257 | 8.978 | 51.457 | 1.708 | 12.202 | 51.457 |
| 5 | .982 | 7.012 | 58.468 |  |  |  |  |  |  |
| 6 | .949 | 6.779 | 65.248 |  |  |  |  |  |  |
| 7 | .884 | 6.313 | 71.561 |  |  |  |  |  |  |
| 8 | .819 | 5.847 | 77.408 |  |  |  |  |  |  |
| 9 | .805 | 5.747 | 83.155 |  |  |  |  |  |  |
| 10 | .767 | 5.477 | 88.632 |  |  |  |  |  |  |
| 11 | .688 | 4.917 | 93.549 |  |  |  |  |  |  |
| 12 | .600 | 4.282 | 97.831 |  |  |  |  |  |  |
| 13 | .287 | 2.050 | 99.881 |  |  |  |  |  |  |
| 14 | .017 | .119 | 100.000 |  |  |  |  |  |  |
